# Use of Semiochemicals for the Management of the Redbay Ambrosia Beetle

**DOI:** 10.3390/insects11110796

**Published:** 2020-11-13

**Authors:** Xavier Martini, Marc A. Hughes, Derrick Conover, Jason Smith

**Affiliations:** 1 North Florida Research and Education Center, Department of Entomology and Nematology, University of Florida, Quincy, FL 32351, USA; dconover13@ufl.edu; 2 Pacific Cooperative Studies Unit, University of Hawai`i at Mānoa, Hilo, HI 96720, USA; mhughes7@hawaii.edu; 3 Institute of Pacific Islands Forestry, USDA Forest Service, Hilo, HI 96720, USA; 4 School of Forest and Resources and Conservation, University of Florida, Gainesville, FL 32611, USA; jasons@ufl.edu

**Keywords:** Scolytinae, forest entomology, Lauraceae, semiochemicals, laurel wilt

## Abstract

**Simple Summary:**

Laurel wilt is a devastating exotic fungal disease that threatens avocado and related members of the laurel family in North America. This disease has killed over 300 million redbay trees and has caused cascading ecological impacts across the landscape. Management strategies, especially in natural forests, are limited. The ambrosia beetles that vector this disease respond strongly to odors produced by the trees, and our research indicates that it is possible to repel the beetles away from trees in a forest setting with the use of verbenone. Other compounds have been identified that can be used to trap the beetles. If used together, these strategies can be used to develop a single “push-pull” system to manage this disease in natural systems where other management strategies are not feasible.

**Abstract:**

This review highlights current advances in the management of the redbay ambrosia beetle, *Xyleborus glabratus*, a primary vector of the pathogenic fungus, *Raffaelea lauricola*, that causes laurel wilt. Laurel wilt has a detrimental effect on forest ecosystems of southeastern USA, with hundreds of millions of Lauraceae deaths. Currently, preventive measures mostly focus on infected-tree removal to potentially reduce local beetle populations and/or use of preventative fungicide applications in urban trees. Use of semiochemicals may offer an opportunity for the management of *X. glabratus*. Research on attractants has led to the development of *α*-copaene lures that are now the accepted standards for *X. glabratus* sampling. Research conducted on repellents first included methyl salicylate and verbenone and attained significant reduction in the number of *X. glabratus* captured on redbay and swamp bay trees treated with verbenone. However, the death rate of trees protected with verbenone, while lower compared to untreated trees, is still high. This work underscores the necessity of developing new control methods, including the integration of repellents and attractants into a single push-pull system.

## 1. Introduction

**Figure 1:**
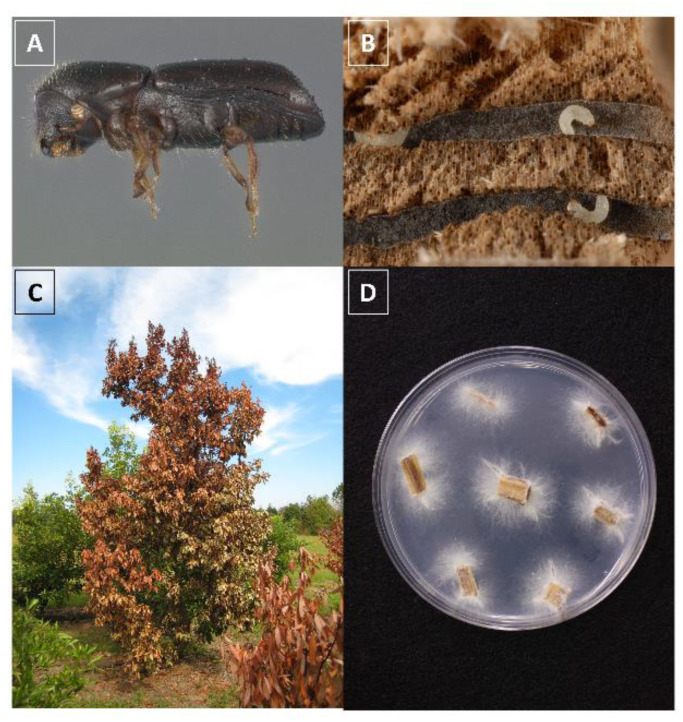
The redbay ambrosia beetles *Xyleborus glabratus* (**A**) adult female and (**B**) larvae within a gallery, (**C**) wilted and recently killed redbay tree and (**D**) *Raffaelea lauricola* in culture. Photo credit (A,B): Lyle Buss, University of Florida.

The invasive redbay ambrosia beetle, *Xyleborus glabratus* Eichhoff (Coleoptera: Scolytinae) (Figure 1) and its symbiont, the fungal pathogen *Raffaelea lauricola* Harrington & Fraedrich was first detected in Georgia in 2002. *Raffaelea lauricola* causes laurel wilt, a deadly vascular disease affecting plants in the Lauraceae in the United States. An ambrosia beetle is either in the sub-family Scolytinae or Platypodinae and has an obligatory association with a fungal symbiont. Typically, these beetles bore a hole into the xylem of the tree, where they excavate a gallery to feed and reproduce. The beetles are considered xylophagous, though they do not actually feed on the tissue of the tree. Instead, the beetles inoculate the tree tissue with their fungal symbiont and feed on the growing spores [1]. Ambrosia beetles in the tribe Xyleborini have a haplodiploid reproduction system; following oviposition, larvae and adults feed on fungal biomass, including mycelial tufts on the gallery walls called sporodochia that consist of clusters of asexual fungal spores (conidia). Foundress females release and propagate fungal symbionts in natal galleries within the tree xylem. Xyleborini males, on the other hand, are flightless and develop from unfertilized eggs. Most male ambrosia beetles are smaller than females and live and die within the galleries [2]. Prior to emerging from the tree to find a new host, adult females will mate with haploid males and collect masses of fungal spores into their mycangia [3].

Introduced into the USA likely from unprocessed wood shipped from Asia [4], *X. glabratus* was first detected in 2002 near Port Wentworth, Georgia [5]. Since then, it is estimated that laurel wilt has been responsible for the death of more than 300 million redbay, *Persea borbonia* (L.) Spreng. trees, which comprises a third of the initial redbay population in US forests [6]. In addition to redbay, other trees in the Lauraceae are impacted by laurel wilt including swamp bay, *P. palustris* (Raf.) Sarg.; silk bay, *P. humilis* Nash; sassafras, *Sassafras albidum* (Nutt.) Nees; the endangered pondberry, *Lindera melissifolia* (Walter) Blume; Gulf licaria, *Licaria triandra* (Sw.) Kosterm.; and pondspice, *Litsea aestivalis* L. [7,8,9,10]. Avocado, *P. americana* Mill. is also susceptible to the disease [11,12]. In this case, however, the fungus is presumably transmitted by an assemblage of different ambrosia beetle species obtaining the spores after lateral transfer from within an infected host [13]. Additionally, infection within groves occurs through root-grafts between trees, leading to infection centers and additional tree mortality without the aid of vector-borne transmission [14].

In a given location, laurel wilt can kill more than 85% of redbay in four years [8,15]. Mortality rates increase with tree size with 100% mortality of trees above 12 cm diameter at breast height in heavily infested sites. The disastrous impact of laurel wilt has important implications for the ecosystem, including those on wildlife that rely on Lauraceae as a food source. On the 178 native arthropod species feeding on North American laurel wilt-susceptible host species, it is estimated that 14% are at high risk of endangerment due to laurel wilt, including 18 species of Lepidoptera such as the palamedes swallowtail butterfly, *Papilio palamedes* Drury (Lepidoptera: Papilionidae), which feeds exclusively on Lauraceae leaves [16,17]. In addition, songbirds, wild turkeys, bobwhite quails, and black bears also feed on the small drupes produced by redbay [18]. While redbay did not disappear from the landscape due to its high propensity to re-sprout from the trunks’ base [15], trees above 15 cm in diameter at breast height can be completely eradicated in areas affected by laurel wilt [19]. The eradication of these trees reduces habitat for nesting birds and increases light penetration, which dramatically alters the herbaceous competition in the forest landscape [19,20,21]. 

In avocado production, the control of laurel wilt is mostly based on the uprooting of infected trees, followed by burning or chipping of infested material [14,22]. The strategy is to decrease the inoculum of the pathogen by reducing/destroying the number of infected trees. This strategy is manageable in avocado groves and nurseries but is difficult to conduct in a natural forest where the location of Lauraceae is rarely known. Other options include entomopathogenic fungi [23] and insecticidal sprays [24]; however, as the females spend most of their life protected within tree trunk and only exit to find a new host, the timing of insecticidal sprays is critical. Fungicide application of propiconazole is also an option and provides protection for 1 year [25]. Alamo^®^ is registered for this purpose for landscape redbay trees, and Tilt^®^ is labeled for this use for commercial avocado. However, both fungicide and insecticide applications are difficult to conduct in a forest setting [26]. In addition, the impact of insecticide applications on non-target species in a forest is an area of concern, even if the sprays are restricted to Lauraceae. Therefore, there is a critical need to develop new tools to control redbay ambrosia beetle and laurel wilt in a forest setting. 

The majority of ambrosia beetles target dying or dead trees and, therefore, contribute to the natural decomposition of wood, as is probably the case for *X. glabratus* in Asia where there is no report that *R. lauricola* causes a widespread plant disease [27]. In the United States, however, females are capable of attacking healthy, presumably unstressed trees, making this species a serious threat for Lauraceae. The infection starts with the initial inoculation with *R. lauricola* by a ‘pioneer’ beetle that bores into the tree sapwood and transfers *R. lauricola* spores contained in the mycangia (specialized sacs located in in the mandibles). However, these pioneer beetles generally fail to develop galleries in the newly infected trees [7]. Next, follows an incubation phase where *X. glabratus* is absent from the wood and *R. lauricola* actively colonizes the tree’s vascular system. The pathogen’s presence in the vascular system rapidly leads to vascular wilt in susceptible hosts (in as little as 14–21 days), and it is believed that this rapid wilt is due to host responses, rather than actions by the pathogen (toxin production etc.) [28]. Host response studies during the host-pathogen interaction of laurel wilt disease development are limited [12,28,29,30,31,32,33]. Time-series studies using electron microscopy of avocado have demonstrated that vascular disruption begins 14 days after fungal inoculation when blockage of vessels occurs (tylosis and presence of gums and gels) [32]. A comparison of laurel wilt responses among avocado cultivars showed significantly lower sapflow, water use efficiencies, carbon assimilation, stomatal conductance of H_2_O, and transpiration in infected vs. mock-infected plants 15 days post-inoculation [28]. Recent work by Adams [29] and Castillo-Argaez [30] confirm with physiological evidence and biochemical analyses that sapflow and transpiration are reduced very early, volatile organic compound production is dramatically altered (within the first few days following infection), and host defense responses are not dependent on extensive pathogen colonization of host tissues.

## 2. Attractants for *X. glabratus*

Since the establishment of *X. glabratus*, a tremendous amount of research has been conducted to develop attractants. The first goal was to improve sampling of *X. glabratus*, and efforts have been highly successful. Contrary to most ambrosia beetles, *X. glabratus* is not attracted to ethanol [34,35], a volatile associated with tree stress and wood decay. Initial studies have unequivocally demonstrated that *X. glabratus* is instead attracted to wood volatiles from redbay and other Lauraceae as bolts cut from redbay trees attracted significantly more *X. glabratus* than bolts cut from non-hosts, and remained attractive to *X. glabratus* for at least 2 months [36]. Further analyses conducted on wood volatiles with gas chromatography and mass spectrometry (GC-MS) analyses determined that Lauraceae wood contains terpenoid compounds including *α*-copaene, *β*-caryophyllene, eucalyptol, *α*-humulene, and cadinene [34,37,38]. Choice test and electroantennography assays determined that *α*-copaene was the compound that elicited the highest attractive response of *X. glabratus* to Lauraceae wood. As *α*-copaene is found in high quantity in manuka oil and phoebe oil [34], these two essential oils became the main attractants used in traps to increase capture of *X. glabratus*. However, phoebe oil quickly became unavailable due to a limited supply and manuka oil showed inconsistent performance [39,40]. Further research identified cubeb oil as a better attractant for *X. glabratus*, until the development of a lure containing up to 50% of *α*-copaene that is currently the most effective for trapping *X. glabratus* [41,42]. 

Lychee wood contains a high quantity of *α*-copaene and attracts a large number of *X. glabratus* [43], despite being a non-host tree for the beetle. Interestingly, when given a choice between lychee wood and avocado wood (which emits less α-copaene), *X. glabratus* preferentially chose lychee wood during the first 4 h of the experiment but later switched to avocado. Further experiments demonstrated that *X. glabratus* does not reproduce in lychee, and the fungus *R. lauricola* does not develop in lychee wood [44]. This indicated that, although *α*-copaene is an important attractant for *X. glabratus*, it is not involved in the boring behavior of the beetle. Conversely, it was found that eucalyptol, a volatile present in wood and leaf volatiles also attracted *X. glabratus* (but at a lower level than *α*-copaene) and increased the boring behavior of *X. glabratus*, [45]. Other compounds found in wood volatiles such as monoterpenes may also have a minor role in *X. glabratus* attraction [38]. 

The reason *X. glabratus* is not considered a pest in Asia but attacks healthy trees in the United States is probably due to an “evolutionary mismatch” as defined by Hulcr et al. [46]. An illustration of this mismatch is that the leaf volatiles play a role in the initial attraction of *X. glabratus* on redbay and swamp bay, even though *X. glabratus* is not capable to develop in healthy trees [38]. Most importantly, *X. glabratus* is attracted to leaf volatiles of redbay and swamp bay but not by leaf volatiles of non-hosts, and this attraction occurs even if the tree is undamaged. This finding is not peculiar, as other wood borers, including bark and ambrosia beetles, may also be attracted to leaf volatiles from their host [47,48]. The profile of redbay leaf volatiles corresponds to the monoterpene portion of the wood volatiles that also contain highly attractive sesquiterpenes [38]. It is possible, therefore, that the initial attraction of *X. glabratus* to a healthy host is driven by the attraction of the beetles to leaf volatiles from redbay, and this attraction to undamaged host leaf volatiles does not occur in Asia. Further studies that compare leaf and wood volatiles from *X. glabratus* native hosts in Asia would be necessary to confirm this hypothesis. 

The symbiotic fungus and laurel wilt pathogen, *R. lauricola*, also releases attractive odors for *X. glabratus* [49,50]. The compounds emitted by *R. lauricola* were identified as ethyl acetate, ethanol, isobutyl alcohol, isoamyl acetate, and isoamyl alcohol [50]. A lure containing a mixture of 36.5: 29: 22: 12.5 of ethyl acetate: ethanol: isoamyl alcohol: isoamyl acetate by volume was tested in a field situation. The lure alone did not appear to attract *X. glabratus* but had a synergistic effect when used with manuka oil [50]. Experiments with more efficient attractive lures such as cubeb oil or *α*-copaene-enriched lures should be conducted to confirm that fungi-emitted volatiles can increase the attraction of *X. glabratus* to Lauraceae wood volatiles. 

The profile of wood tissue volatiles of redbay does not change following infection with *R. lauricola*. In the early investigation by Hanula et al. [36], the authors found no increase of *X. glabratus* capture when a laurel wilt-infected wood bolt was used as a lure compared to an uninfected bolt lure. These results were confirmed in olfactometer assays and through GC-MS analysis [33]. Infected wood volatiles were similar to those of uninfected wood, and there were no significant increases in beetle attraction to infected wood compared to uninfected wood in olfactometer tests. Interestingly, Fraedrich et al. [51] found that *X. glabratus* does produce more abundant brood in bolts colonized with *R. lauricola* than from healthy trees and hypothesized that physical properties in diseased sapwood could be more conducive to brood productions. 

## 3. Repellents against *X. glabratus*

**Figure 2:**
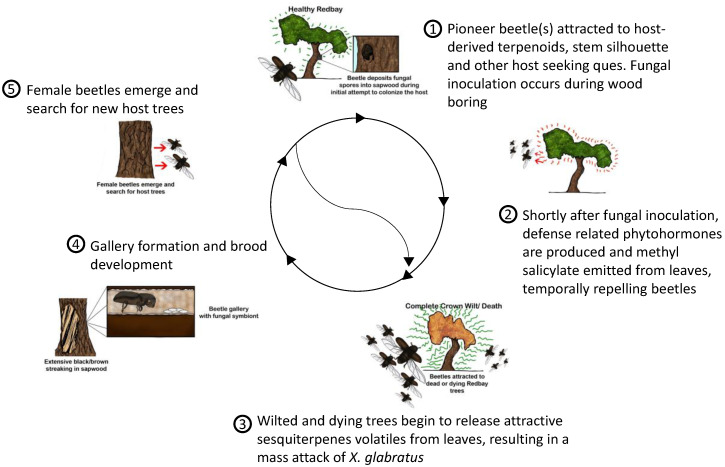
Cycle of the redbay ambrosia beetle *Xyleborus glabratus* in relation to disease progression in redbay.

Contrary to wood volatiles, sequential changes in leaf volatiles were detected in *R. lauricola*-infected trees [33]. Quickly after infection with *R. lauricola*, the Lauraceae host responds with high emission of methyl salicylate in leaf volatiles. Increased methyl salicylate production is an indication of the activation of the salicylic acid pathway involved in systemic acquired resistance [52]. In redbay, the increase of methyl salicylate emissions is coupled with an increase of salicylic acid and h-indole-3-propionic acid in leaf tissues, two phytohormones associated with systemic acquired resistance. As the disease progresses within the tree and leaves begin wilting, methyl salicylate decreases and sesquiterpenes, including α-copaene, that are normally absent from non-infected host leaf volatiles are emitted through the leaves [33]. These changes induce a different response from *X. glabratus* compared to uninfected trees. The beetles avoid leaf volatiles of trees recently infected and emitting methyl salicylate. Complementary olfactometer tests demonstrated that methyl salicylate was repellent to *X. glabratus* and induced avoidance of infected trees the first weeks following infection. This is in accordance with the observation that *X. glabratus* are absent from wood tissues following the initial inoculation of the fungus [7]. In the second phase, the decrease in methyl salicylate emission coupled with the increase in sesquiterpene emission in leaf volatiles was associated with attraction of *X. glabratus*. The sequential changes in leaf volatiles following infection with *R. lauricola* offers a plausible explanation regarding the absence of beetles following infection and the attraction of conspecifics at the appearance of the first laurel wilt symptoms (Figure 2).

**Figure 3:**
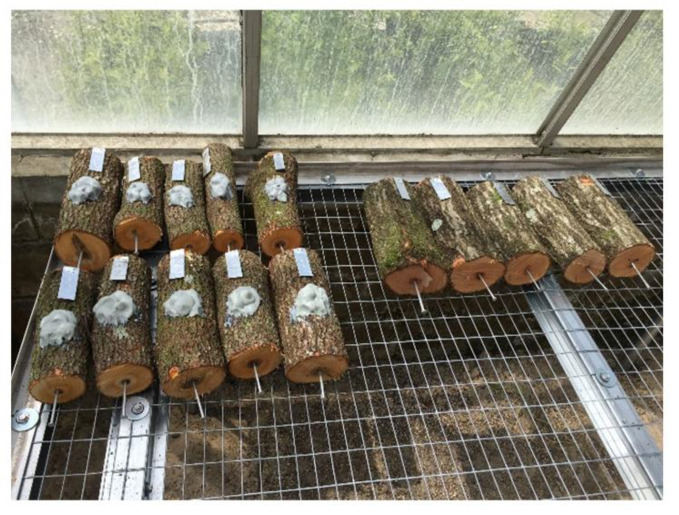
Freshly cut redbay bolts baited with SPLAT and non-baited controls used in the seminal repellent study in Hughes et al. (2017).

**Figure 4:**
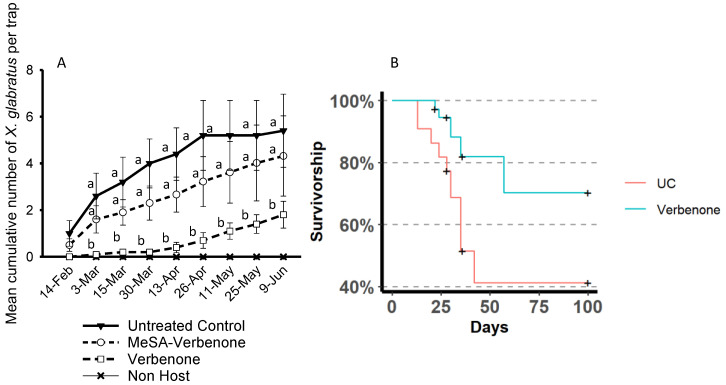
(**A**)**:** Mean (±SE) of cumulative capture of *Xyleborus glabratus* during field trials in Wakulla Spring State Park (FL), following a single application of verbenone and a mixture of verbenone and methyl salicylate (MeSA-Verbenone). Points labeled by different lower-case letters within treatments are significantly different at the *p* < 0.05 level for the given date. From Martini et al. (2020). (**B**) Cumulated survivorship of redbay and swamp bay trees treated with verbenone on four different studies in Wakulla State Park, Ichetucknee Springs State Park, Historic Hail Homestead Lake and Lake Talquin State Park (FL), conducted from 2017 to 2018. UC (untreated control) and verbenone treatment differed significantly at the *p* < 0.05. Crosses indicate censured data.

As methyl salicylate is an inexpensive and easily available compound, its use in field situations has been evaluated in two successive studies. In the first study, tests were conducted on redbay cut bolts disposed in a forest setting. Methyl salicylate was integrated within a specialized pheromone and lure application technology (SPLAT) matrix (ISCA Technologies, Riverside, California, USA) (Figure 3). The number of beetles captured on the traps attached to each redbay bolt, as well as the holes bored by the beetles were compared. In this study, methyl salicylate successfully reduced the number of *X. glabratus* on the traps compared to controls, and, more importantly, decreased the number of boring holes resulting from the beetle attacks [53]. However, in a subsequent study, the experiments on whole swamp bay trees were largely unsuccessful [54] (Figure 4A). The reasons why tests of methyl salicylate were positive in olfactometer assays and on bolts but not on whole trees are unclear; however, it is obvious that the overall area to protect on a whole tree is larger than on a bolt, and it is possible that higher methyl salicylate emission rates are needed to protect the trees. The method of methyl salicylate application might also be a reason for the failed attempt to repel *X. glabratus* on whole trees. Methyl salicylate repellent was applied on the trunk, while methyl salicylate is released by the leaves in infected trees. By applying methyl salicylate on the trunk, the shape of the plume of methyl salicylate released by the leaves may be altered. An area of future research would be to stimulate the salicylic acid pathway in redbay with the exogenous application of salicylic acid or a salicylic acid analogue to stimulate the release of methyl salicylate. Manipulation using salicylic acid sprays has been successful in citrus [55], where the authors were able to stimulate an increase of methyl salicylate from 1.1% to 65% of the total volatile blend emitted by citrus leaves. 

In the search for repellents against redbay ambrosia beetles better results have been obtained with verbenone. Some major bark beetle pests such as the mountain pine beetle have been successfully controlled by verbenone in the past [56,57,58,59]. Verbenone was successful when integrated with SPLAT [53,54]. SPLAT Verb^®^ is a wax-like matrix that can be applied directly on the trunk with a caulking gun and releases volatiles over time (Figure 3). Tests on redbay bolts and whole trees showed a significant decrease in the number of *X. glabratus* that landed on redbay trees. The dosage was four 17.5 g dollops (70 g total) of SPLAT Verb^®^ per tree. This corresponds approximately to the emission of 70 mg of verbenone per day on the whole tree. It resulted in a significant decrease in the number of *X. glabratus* beetles captured per tree. In the study we conducted in the forest in north and central Florida, we were able to obtain a repellent effect for four months with a single application of SPLAT. On redbay logs, the decrease in beetle captures on traps was associated with a decrease in boring holes, which is important as it is during boring that the beetles transmit the fungus. In term of tree survival, the aggregation of tree survival from five field trials conducted in 2017 and 2018 (including the three published in Martini et al. [54]), in four different locations in Florida revealed that treatment with verbenone increased significantly the survivorship of redbay and swamp bay in forests with endemic presence of *X. glabratus* from 41.2 to 70.2% after 100 days (Cox Model: z = −2.381, n = 59, *p* = 0.0173) (Figure 4B). 

## 4. Moving to a Push-Pull System

The primary question is how to improve the efficacy of verbenone in the field. A logical option would be to integrate verbenone within a push-pull system. A push-pull system combines a repellent force (“push”) with an attractant force (“pull”) [60,61]. In our case, both the attractant (*α*-copaene lures) and the repellent (verbenone) are identified. Applying a push-pull strategy to control Scolytinae beetles has been conducted successfully in other systems. Using verbenone and attractive pheromone blends, a reduction in the number of attacks by the mountain pine beetle on lodgepole pine was achieved in British Columbia [62]. Similarly, a push-pull strategy deployed in California and Washington state, using verbenone and green leaf volatiles as attractants significantly reduced the attacks of mountain pine beetles on lodgepole pine and whitebark pine [63]. However, a push-pull strategy using ethanol traps and verbenone as attractants and repellents, respectively, failed to reduce non-native ambrosia beetle attacks on potted dogwoods [64]. In avocado, where *R. lauricola* is likely transmitted by beetles other than *X. glabratus* [65], a push-pull strategy using ethanol (that attracts most beetles found in avocado groves) and verbenone showed promising results [66]: the addition of attractive lures had a significant interaction with verbenone, and no ambrosia beetles were captured within the push-pull treatment on traps situated less than 1 m from the treated trees during the duration of the experiment (30 days). 

The possibility that verbenone and *α*-copaene may interact and decrease *X. glabratus* settling more than verbenone alone has to be explored. In addition to semiochemical attractants, it is possible that visual cues may also play an important role. As *X. glabratus* often use tree silhouettes to find their host [67], the use of a visual attractant mimicking tree silhouette baited with α-copaene, combined with verbenone may be a pertinent area of research.

## 5. Conclusions

Since the arrival of *X. glabratus*, extensive research has been conducted on the chemical ecology of the beetle. Significant progress has been made on the attractant, leading to *α*-copaene-enriched lures, which are currently the most effective for trapping the beetles. Work on repellents has recently shown significant progress with reproducible positive results using verbenone. However, the death rate of redbay in forests is still high despite the protection of verbenone. More holistic strategies incorporating attractants and repellents are needed to protect redbay in forest settings. 

## References

[1] Kasson M.T., Wickert K.L., Stauder C.M., Macias A.M., Berger M.C., Dylan D.R.S., Short P.G., DeVallance D.B., Hulcr J. (2016). Mutualism with aggressive wood-degrading *Flavodon ambrosius* (Polyporales) facilitates niche expansion and communal social structure in Ambrosiophilus ambrosia beetles. Fungal Ecol..

[2] Cognato A.I., Hulcr J., Dole S.A., Jordal B.H. (2011). Phylogeny of haplo-diploid, fungus-growing ambrosia beetles (Curculionidae: Scolytinae: Xyleborini) inferred from molecular and morphological data. Zool. Scr..

[3] Hulcr J., Stelinski L.L. (2017). The ambrosia symbiosis: From evolutionary ecology to practical management. Annu. Rev. Entomol..

[4] Harrington T.C., Yun H.Y., Lu S.-S., Goto H., Aghayeva D.N., Fraedrich S.W. (2011). Isolations from the redbay ambrosia beetle, *Xyleborus glabratus*, confirm that the laurel wilt pathogen, *Raffaelea lauricola*, originated in Asia. Mycologia.

[5] Fraedrich S.W., Harrington T.C., Rabaglia R.J. (2007). Laurel wilt: A new and devastating disease of redbay caused by a fungal symbiont of the exotic redbay ambrosia beetle. Newsl. Mich. Entomol. Soc..

[6] Hughes M.A., Riggins J.J., Koch F.H., Cognato A.I., Anderson C., Formby J.P., Dreaden T.J., Ploetz R.C., Smith J.A. (2017). No rest for the laurels: Symbiotic invaders cause unprecedented damage to southern USA forests. Biol. Invasions.

[7] Fraedrich S.W., Harrington T.C., Rabaglia R.J., Ulyshen M.D., Mayfield A.E., Hanula J.L., Eickwort J.M., Miller D.R. (2008). A fungal symbiont of the redbay ambrosia beetle causes a lethal wilt in redbay and other Lauraceae in the Southeastern United States. Plant Dis..

[8] Ploetz R.C., Konkol J. (2013). First report of gulf licaria, *Licaria trianda*, as a suscept of laurel wilt. Plant Dis..

[9] Smith J.A., Dreaden T.J., Mayfield Iii A.E., Boone A., Fraedrich S.W., Bates C. (2009). First report of laurel wilt disease caused by *Raffaelea lauricola* on sassafras in Florida and South Carolina. Plant Dis..

[10] Hughes M., Smith J.A., Mayfield A.E., Minno M.C., Shin K. (2011). First report of laurel wilt disease caused by *Raffaelea lauricola* on pondspice in Florida. Plant Dis..

[11] Evans E.A., Crane J., Hodges A., Osborne J.L. (2010). Potential economic impact of laurel wilt disease on the Florida avocado industry. Horttechnology.

[12] Ploetz R.C., Perez-Martinez J., Smith J.A., Hughes M.A., Dreaden T.J., Inch S.A., Fu Y. (2012). Responses of avocado to laurel wilt, caused by *Raffaelea lauricola*. Plant Pathol..

[13] Carrillo D., Duncan R.E., Ploetz J.N., Campbell A.F., Ploetz R.C., Peña J.E. (2014). Lateral transfer of a phytopathogenic symbiont among native and exotic ambrosia beetles. Plant Pathol..

[14] Ploetz R.C., Hughes M.A., Kendra P.E., Fraedrich S.W., Carrillo D., Stelinski L.L., Hulcr J., Mayfield A.E.I., Dreaden T.J., Crane J.H. (2017). Recovery plan for laurel wilt of avocado, caused by *Raffaelea lauricola*. Plant Health Prog..

[15] Cameron R., Hanula J., Fraedrich S., Bates C. (2015). Progression and impact of laurel wilt disease within redbay and sassafras populations in Southeast Georgia. Southeast. Nat..

[16] Chupp A.D., Battaglia L.L. (2014). Potential for host shifting in *Papilio palamedes* following invasion of laurel wilt disease. Biol. Invasions.

[17] Riggins J.J., Chupp A.D., Formby J.P., Dearing N.A., Bares H.M., Brown R.L., Oten K.F. (2019). Impacts of laurel wilt disease on arthropod herbivores of North American Lauraceae. Biol. Invasions.

[18] Brendemuehl R.H., Burns R.M., Honkala L.H. (1990). Persea borbonia (L.) Spreng. Redbay. Silvics of North America.

[19] Spiegel K.S., Leege L.M. (2013). Impacts of laurel wilt disease on redbay (*Persea borbonia* (L.) Spreng.) population structure and forest communities in the coastal plain of Georgia, USA. Biol. Invasions.

[20] Goldberg N., Heine J. (2009). A comparison of arborescent vegetation pre- (1983) and post- (2008) outbreak of the invasive species the Asian ambrosia beetle *Xyleborus glabratus* in a Florida maritime hammock. Plant Ecol. Divers..

[21] Evans J.P., Scheffers B.R., Hess M. (2014). Effect of laurel wilt invasion on redbay populations in a maritime forest community. Biol. Invasions.

[22] Hughes M.A., Smith J.A., Ploetz R.C., Kendra P.E., Mayfield A., Hanula J., Hulcr J., Stelinski L.L., Cameron S., Riggins J.J. (2015). Recovery plan for laurel wilt on redbay and other forest species caused by *Raffaelea lauricola* and disseminated by *Xyleborus glabratus*. Plant Health Prog..

[23] Carrillo D., Dunlap C.A., Avery P.B., Navarrete J., Duncan R.E., Jackson M.A., Behle R.W., Cave R.D., Crane J.H., Rooney A.P. (2015). Entomopathogenic fungi as biological control agents for the vector of the laurel wilt disease, the redbay ambrosia beetle, *Xyleborus glabratus* (Coleoptera: Curculionidae). Biol. Control.

[24] Carrillo D., Crane J.H., Peña J.E. (2013). Potential of contact insecticides to control *Xyleborus glabratus* (Coleoptera: Curculionidae), a vector of laurel wilt disease in avocados. J. Econ. Entomol..

[25] Ploetz R.C., Pérez-Martínez J.M., Evans E.A., Inch S.A. (2011). Toward fungicidal management of laurel wilt of avocado. Plant Dis..

[26] Gitau C.W., Bashford R., Carnegie A.J., Gurr G.M. (2013). Review: A review of semiochemicals associated with bark beetle (Coleoptera: Curculionidae: Scolytinae) pests of coniferous trees: A focus on beetle interactions with other pests and their associates. For. Ecol. Manag..

[27] Hulcr J., Lou Q.-Z. (2013). The redbay ambrosia beetle (Coleoptera: Curculionidae) prefers Lauraceae in its native range: Records from the Chinese National Insect Collection. Fla. Entomol..

[28] Ploetz R., Schaffer B., Vargas A., Konkol J., Salvatierra J., Wideman R. (2015). Impact of laurel wilt, caused by *Raffaelea lauricola*, on leaf gas exchange and xylem sap flow in avocado, Persea americana. Phytopathology.

[29] Adams S.A. (2020). Effects of Paclobutrazol on the Raffaelea Lauricola-Persea Pathosystem.

[30] Castillo-Argaez R., Schaffer B., Vazquez A., Sternberg L.D.S.L. (2020). Leaf gas exchange and stable carbon isotope composition of redbay and avocado trees in response to laurel wilt or drought stress. Environ. Exp. Bot..

[31] Inch S.A., Ploetz R.C. (2012). Impact of laurel wilt, caused by *Raffaelea lauricola*, on xylem function in avocado, Persea americana. For. Pathol..

[32] Inch S., Ploetz R., Held B., Blanchette R. (2012). Histological and anatomical responses in avocado, *Persea americana*, induced by the vascular wilt pathogen. Botany.

[33] Martini X., Hughes M.A., Killiny N., George J., Lapointe S.L., Smith J.A., Stelinski L.L. (2017). The fungus *Raffaelea lauricola* modifies behavior of its symbiont and vector, the redbay ambrosia beetle (*Xyleborus glabratus*), by altering host plant volatile production. J. Chem. Ecol..

[34] Hanula J.L., Sullivan B. (2008). Manuka oil and phoebe oil are attractive baits for *Xyleborus glabratus* (Coleoptera: Scolytinae ), the vector of laurel wilt. Environ. Entomol..

[35] Johnson C., Cameron R., Hanula J., Bates C. (2014). The attractiveness of manuka oil and ethanol, alone and in combination, to *Xyleborus glabratus* (Coleoptera: Curculionidae: Scolytinae) and other Curculionidae. Fla. Entomol..

[36] Hanula J.L., Mayfield A.E., Fraedrich S.W., Rabaglia R.J. (2008). Biology and host associations of redbay ambrosia beetle (Coleoptera: Curculionidae: Scolytinae), exotic vector of laurel wilt killing redbay trees in the southeastern United States. J. Econ. Entomol..

[37] Niogret J., Kendra P.E., Epsky N.D., Heath R.R. (2011). Comparative analysis of terpenoid emissions from Florida host trees of the redbay ambrosia beetle, *Xyleborus glabratus* (Coleoptera: Curculionidae: Scolytinae). Fla. Entomol..

[38] Martini X., Hughes M.A., Smith J.A., Stelinski L.L. (2015). Attraction of redbay ambrosia beetle, *Xyleborus glabratus*, to leaf volatiles of its host plants in North America. J. Chem. Ecol..

[39] Kendra P.E., Montgomery W.S., Niogret J., Schnell E.Q., Deyrup M.A., Epsky N.D. (2014). Evaluation of seven essential oils identifies cubeb oil as most effective attractant for detection of *Xyleborus glabratus*. J. Pest. Sci..

[40] Kendra P.E., Montgomery W.S., Niogret J., Tabanca N., Owens D., Epsky N.D. (2018). Utility of essential oils for development of host-based lures for *Xyleborus glabratus* (Coleoptera: Curculionidae: Scolytinae), vector of laurel wilt. Open Chem..

[41] Kendra P.E., Montgomery W.S., Deyrup M.A., Wakarchuk D. (2015). Improved lure for redbay ambrosia beetle developed by enrichment of α-copaene content. J. Pest Sci..

[42] Kendra P.E., Niogret J., Montgomery W.S., Deyrup M.A., Epsky N.D. (2015). Cubeb oil lures: Terpenoid emissions, trapping efficacy, and longevity for attraction of redbay ambrosia beetle (Coleoptera: Curculionidae: Scolytinae). J. Econ. Entomol..

[43] Kendra P.E., Montgomery W.S., Niogret J., Peña J.E., Capinera J.L., Brar G., Epsky N.D., Heath R.R. (2011). Attraction of the redbay ambrosia beetle, *Xyleborus glabratus*, to avocado, lychee, and essential oil Lures. J. Chem. Ecol..

[44] Kendra P.E., Ploetz R.C., Montgomery W.S., Peña J.E., Brar G.S., Epsky N.D. (2013). Evaluation of *Litchi chinensis* for host status to *Xyleborus glabratus* (Coleoptera: Curculionidae: Scolytinae) and susceptibility to laurel wilt disease. Fla. Entomol..

[45] Kuhns E.H., Martini X., Tribuiani Y., Coy M., Gibbard C., Peña J., Hulcr J., Stelinski L.L. (2014). Eucalyptol is an attractant of the redbay ambrosia beetle, *Xyleborus glabratus*. J. Chem. Ecol..

[46] Hulcr J., Black A., Prior K., Chen C.-Y., Li H.-F. (2017). Studies of ambrosia beetles (Coleoptera: Curculionidae) in their native ranges help predict invasion impact. Fla. Entomol..

[47] Pham D.L., Ito Y., Okada R., Ikeno H., Isagi Y., Yamasaki M. (2019). Effects of leaf conditions and flight activity on the behaviour of *Platypus quercivorus* (Murayama) (Coleoptera: Platypodidae). J. Appl. Entomol..

[48] Peterson D.L., Böröczky K., Tumlinson J., Cipollinia D. (2020). Ecological fitting: Chemical profiles of plant hosts provide insights on selection cues and preferences for a major buprestid pest. Phytochemistry.

[49] Hulcr J., Mann R., Stelinski L.L. (2011). The scent of a partner: Ambrosia beetles are attracted to volatiles from their fungal symbionts. J. Chem. Ecol..

[50] Kuhns E.H., Tribuiani Y., Martini X., Meyer W.L., Peña J., Hulcr J., Stelinski L.L. (2014). Volatiles from the symbiotic fungus *Raffaelea lauricola* are synergistic with manuka lures for increased capture of the redbay ambrosia beetle *Xyleborus glabratus*. Agric. For. Entomol..

[51] Fraedrich S.W., Harrington T.C., Huang Q., Zarnoch S.J., Hanula J.L., Best G.S. (2018). Brood production by *Xyleborus glabratus* in bolts from trees infected and uninfected with the laurel wilt pathogen. Raffaelea lauricola. For. Sci..

[52] Park S.W., Kaimoyo E., Kumar D., Mosher S., Klessig D.F. (2007). Methyl salicylate is a critical mobile signal for plant systemic acquired resistance. Science.

[53] Hughes M.A., Martini X., Kuhns E., Colee J., Mafra-Neto A., Stelinski L.L., Smith J.A. (2017). Evaluation of repellents for the redbay ambrosia beetle, *Xyleborus glabratus*, vector of the laurel wilt pathogen. J. Appl. Entomol..

[54] Martini X., Sobel L., Conover D., Mafra-Neto A., Smith J. (2020). Verbenone reduces landing of the redbay ambrosia beetle, vector of the laurel wilt pathogen, on live standing redbay trees. Agric. For. Entomol..

[55] Patt J.M., Robbins P.S., Niedz R., McCollum G., Alessandro R. (2018). Exogenous application of the plant signalers methyl jasmonate and salicylic acid induces changes in volatile emissions from citrus foliage and influences the aggregation behavior of Asian citrus psyllid (*Diaphorina citri*), vector of Huanglongbing. PLoS ONE.

[56] Shea P.J., McGregor M.D., Daterman G.E. (1992). Aerial application of verbenone reduces attack of Lodgepole pine by mountain pine-beetle. Can. J. For. Res..

[57] Bentz B.J., Kegley S., Gibson K., Thier R. (2005). A test of high-dose verbenone for stand-level protection of lodgepole and whitebark pine from mountain pine beetle (Coleoptera: Curculionidae: Scolytinae) attacks. J. Econ. Entomol..

[58] Gillette N.E., Stein J.D., Owen D.R., Webster J.N., Fiddler G.O., Mori S.R., Wood D.L. (2006). Verbenone-releasing flakes protect individual *Pinus contorta* trees from attack by *Dendroctonus ponderosae* and *Dendroctonus valens* (Coleoptera: Curculionidae, Scolytinae). Agric. For. Entomol..

[59] Perkins D., Jorgensen C., Rinella M. (2015). Verbenone decreases whitebark pine mortality throughout a mountain pine beetle outbreak. For. Sci..

[60] Cook S.M., Khan Z.R., Pickett J.A. (2007). The use of push-pull strategies in integrated pest management. Annu. Rev. Entomol..

[61] Eigenbrode S.D., Birch A.N.E., Lindzey S., Meadow R., Snyder W.E. (2016). A mechanistic framework to improve understanding and applications of push-pull systems in pest management. J. Appl. Ecol..

[62] Borden J.H., Birmingham A.L., Burleigh J.S. (2006). Evaluation of the push-pull tactic against the mountain pine beetle using verbenone and non-host volatiles in combination with pheromone-baited trees. For. Chron..

[63] Gillette N.E., Mehmel C.J., Mori S.R., Webster J.N., Wood D.L., Erbilgin N., Owen D.R. (2012). The push-pull tactic for mitigation of mountain pine beetle (Coleoptera: Curculionidae) damage in lodgepole and whitebark pines. Environ. Entomol..

[64] Werle C.T., Ranger C.M., Schultz P.B., Reding M.E., Addesso K.M., Oliver J.B., Sampson B.J. (2018). Integrating repellent and attractant semiochemicals into a push-pull strategy for ambrosia beetles (Coleoptera: Curculionidae). J. Appl. Entomol..

[65] Carrillo D., Duncan R.E., Peña J.E. (2012). Ambrosia beetles (Coleoptera: Curculionidae: Scolytinae) that breed in avocado wood in Florida. Fla. Entomol..

[66] Rivera M.J., Martini X., Conover D., Mafra-Neto A., Carrillo D., Stelinski L.L. (2020). Evaluation of semiochemical based push-pull strategy for populatio suppression of ambrosia beetle vectors of laurel wilt disease in avocado. Sci. Rep..

[67] Mayfield A.E., Brownie C. (2013). The redbay ambrosia beetle (Coleoptera: Curculionidae: Scolytinae) uses stem silhouette diameter as a visual host-finding cue. Environ. Entomol..

